# Human amnion-derived mesenchymal stem cells promote osteogenic and angiogenic differentiation of human adipose-derived stem cells

**DOI:** 10.1371/journal.pone.0186253

**Published:** 2017-10-11

**Authors:** Chunli Zhang, Lidong Yu, Songjian Liu, Yuli Wang

**Affiliations:** 1 Department of Clinical Research, Friendship Plastic Surgery Hospital, Nanjing Medical University, Nanjing, Jiangsu, The People’s Republic of China; 2 Department of Plastic Surgery, The Second Affiliated Hospital of Nanjing Medical University, Nanjing, Jiangsu, The People’s Republic of China; 3 Department of Plastic Surgery, The First Affiliated Hospital of Nanjing Medical University, Nanjing, Jiangsu, The People’s Republic of China; 4 Jiangsu Key Laboratory of Oral Diseases, Nanjing Medical University, Nanjing, Jiangsu, The People’s Republic of China; Instituto Butantan, BRAZIL

## Abstract

Tissue engineering using suitable mesenchymal stem cells (MSCs) shows great potential to regenerate bone defects. Our previous studies have indicated that human amnion-derived mesenchymal stem cells (HAMSCs) could promote the osteogenic differentiation of human bone marrow mesenchymal stem cells (HBMSCs). Human adipose-derived stem cells (HASCs), obtained from adipose tissue in abundance, are capable of multi-lineage differentiation. In this study, the effects of HAMSCs on osteogenic and angiogenic differentiation of HASCs were systematically investigated. Proliferation levels were measured by flow cytometry. Osteoblastic differentiation and mineralization were investigated using chromogenic alkaline phosphatase activity (ALP) activity substrate assays, Alizarin red S staining, real-time polymerase chain reaction (real-time PCR) analysis of osteogenic marker expression, and Western blotting. We found that HAMSCs increased the proliferation and osteoblastic differentiation of HASCs. Moreover, enzyme-linked immunosorbent assay (ELISA) and human umbilical vein endothelial cells (HUVECs) tube formation suggested HAMSCs enhanced angiogenic potential of HASCs via secretion of increased vascular endothelial growth factor (VEGF). Thus, we conclude that HAMSC might be a valuable therapeutic approach to promote HASCs-involved bone regeneration.

## Introduction

Effective reconstruction of bone defects resulting from trauma and surgical resection is becoming a major clinical challenge in maxillofacial surgery [1–3]. With the development of regenerative medicine, tissue engineering utilizing optimal scaffolds seeded with mesenchymal stem cells has shown a promising potential in generating new bone tissue[4, 5]. Human adipose-derived stem cells (HASCs) can be obtained from adipose, a highly abundant tissue and easily accessible pool of stem cells [6–8]. HASCs are capable of self-renewal and differentiation into several distinct cell lineages[9]. HASCs are known to present good bone regenerative capacity [10]. Besides, the pericyte-like phenotype formed by HASCs has been shown to play a role in blood vessels maturation and remodeling as an autologous cell source [11–13]. By far, gene therapy, cytokines and Chinese herb extracts have been used to enhance the osteogenic and angiogenic differentiation of HASCs[14–16]. However, disadvantages such as individual differences and easy degradation limit their application.

Human amnion-derived mesenchymal stem cells (HAMSCs) can be obtained from human term placenta, a highly abundant tissue and valuable source of stem/progenitor cells[17]. HAMSCs have potential of differentiation into several cell types including bone, cartilage, fat, and muscle[18].They are associated with low anti-inflammatory properties and fewer ethical issues than other sources of stem cells, thus providing considerable benefits in bone tissue engineering [19]. Our previous study found that HAMSCs were capable of providing a conducive environment to drive osteogenic differentiation of human bone marrow mesenchymal stem cells (HBMSCs)[20]. Moreover, HAMSCs are able to augment blood perfusion and increase intraneural vascularity[21], thus possessing higher angiogenic property compared to HASCs[22]. Considering the excellent osteogenic and angiogenic ability of HAMSCs, we hypothesize that an appropriate cell-cell culture system may effectively promote both osteogenesis and angiogenesis in HASCs. In the present study, we used a transwell coculture system to evaluate the *in vitro* effects of HAMSCs on the proliferation, osteogenic and angiogenic differentiation of HASCs. Moreover, the role of potential signal pathways involved in this process was also investigated.

## Materials and methods

### Chemicals and reagents

Trypsin-ethylenediaminetetraacetic acid (EDTA), fetal bovine serum (FBS) and phosphate-buffered saline (PBS) were purchased from Gibco^®^ Life Technologies. The Alizarin red S (pH 4.4), protein assay kit, lysis buffer, ALP and bicinchoninic acid (BCA) assay kits were purchased from the Jiancheng Corp (Nanjing, China). EBM (endothelial basal medium) was purchased from ScienCell^®^ (San Diego, USA). Penicillin G-streptomycin sulfate, α-minimum essential medium (αMEM), dexamethasone, β-glycerophosphate ascorbic acid and dimethyl sulfoxide (DMSO) were purchased from Sigma–Aldrich (St. Louis, MO). Six-well culture plates and transwells (6-Well Millicell Hanging Cell Culture Inserts, 0.4 μm, PET) were purchased from Millipore® (Bedford, MA, USA). Human VEGF ELISA kit was purchased from R&D Systems (Minneapolis, MN, USA). Growth factor-reduced Matrigel was purchased from BD Bioscience (San Diego, CA). EGM-2 BulletKit was purchased from Lonza (Walkersville, MD, USA). The mouse anti-rabbit IgG (L27A9) mAb, anti-mouse IgG (HRP-linked Antibody #7076), phospho-p44/42 (p-ERK1/2) MAPK rabbit mAb, p44/42 MAPK (ERK1/2) rabbit mAb, phospho-p38 (p-p38) MAPK (Thr180/Tyr182) (D3F9) rabbit mAb, p38 MAPK (D13E1) rabbit mAb, RUNX2 (D1L7F) rabbit mAb, phospho-SAPK/JNK (p-JNK) (Thr183/Tyr185) (81E11) Rabbit mAb, SAPK/JNK (JNK) antibody (#9252),and β-Actin (13E5) Rabbit mAb were purchased from Cell Signalling Technology. The anti-Osteocalcin (OCN) antibody (ab133612), anti-Collagen I (COL1) antibody (ab6308), anti-VEGF Receptor 1 (VEGFR1) antibody (ab32152), and anti-Angiogenin (ab10600) were purchased from Abcam. Other reagents used were of the highest commercial grade available.

### Cell culture

HASCs were obtained from the American Type Culture Collection (ATCC, Manassas, VA, USA). HUVECs were purchased from the China Infrastructure of Cell Line Resources (Beijing, China). Isolation of HAMSCs was performed following the pancreatin/collagenase digestion method[23–25]. HASCs and HAMSCs were cultured in 60-mm plates in αMEM supplemented with 100 U/L penicillin, 100 mg/L streptomycin, and 10% FBS in a humidified atmosphere of 5% CO2 at 37°C. HUVECs were cultured in 60-mm plates in EBM containing 1% FBS and EGM-2 BulletKit in a humidified atmosphere of 5% CO2 at 37°C. Cells from passage 3 were used and culture medium was changed every 3 days. The study protocols were approved by the Ethics Committee of the School of Stomatology, Nanjing Medical University, China (NO.PJ2013-037-001). Informed consent was obtained from all the participants enrolled in this study.

### The coculture system

A transwell coculture system was used to investigate the effects of HAMSCs on HASCs as described previously[26]. HASCs were seeded at an initial cell density of 5×10^4^ cells/cm^2^ in 6-well culture plates. Transwells were placed in other 6-well culture plates and HAMSCs were seeded at increasing HASCs: HAMSCs ratios (5×10^4^cells/transwell, 10×10^4^cells/transwell and 15×10^4^cells/transwell). HASCs were subjected to a 24 h treatment with serum-free medium following the attachment of the cells (approximately 12 h). After washing with PBS, transwells containing HAMSCs were transferred into the corresponding wells of the 6-well culture plate containing HASCs to create the HASCs/HAMSCs transwell coculture system. HASCs in wells with transwells served as the treatment groups, while HASCs without transwells were used as the control groups.

### Flow cytometry

The effects of HAMSCs on HASCs proliferation was measured by flow cytometry at 1, 3 and 5 days. Briefly, after starvation in serum-free medium for 24 h, HASCs were washed with PBS. Transwells containing HAMSCs were moved into the corresponding wells of the 6-well culture plate containing HASCs, and the medium was replaced with culture medium containing 10% FBS. HASCs were harvested at day 1, 3 and 5 and fixed with 75% ice-cold ethanol at 4°C for 30 min in the dark. DNA content was measured by a FACScan flow cytometer (BD Biosciences, Franklin Lakes, NJ, USA) and the cell cycle fractions (G0, G1, S, and G2 M phases) were processed using CellQuest Pro software (BD Biosciences). Data was analyzed by ModFitLT 3.2 (verity software house, USA).

### ALP activity and mineralized matrix formation

After transwells containing HAMSCs were moved into the corresponding wells of the 6-well culture plate containing HASCs, both the cell types were cultured in osteogenic medium (OS) containing 10 mM β-glycerophosphate, 100 nM ascorbic acid, and 100 nM dexamethasone. HAMSCs, HASCs, and HASCs/HAMSCs groups were subjected to ALP activity assays and Alizarin red staining. ALP activity assay was performed using an ALP assay kit according to the manufacturer’s protocols at 7 and 14 days. Alizarin red staining was performed at day 21 using 40 mM Alizarin red S (pH 4.4) for 10 min at room temperature. Following rinsing with PBS, mineralized nodules were visualized using an inverted microscope (Carl Zeiss AG, Oberkochen, Germany) and 10 images were captured for each group.

### Total RNA extraction and real-time PCR

Total RNA was isolated from HASCs in the control and treatment groups by using trizol reagent, according to the manufacturer’s instructions. The RNA was reverse-transcribed into cDNA in a 20-μL reaction by using a PrimeScript RT Master Mix kit. Real-time reverse transcription-PCR was performed with a SYBR Green PCR kit (Toyobo, Osaka, Japan) and ABI 7300 Real-time PCR System (Applied Biosystems; Thermo Fisher Scientific, Inc.). Specific primers were designed as follows: human RUNX2 (forward, 5’-CCGCACAACCGCACCAT-3’; reverse, 5’-CGCTCCGGCCCACAAATCTC-3’), human OCN (forward, 5’-CATGAGAGCCCTCACA-3’; reverse, 5’-AGAGCGACACCCTAGAC-3’),human COL1 (forward, 5’-GGACACAATGGATTGCAAGG-3’; reverse, 5’-TAACCACTGCTCCACTCTGG-3’), human VEGF (forward, 5’-TAGTCGACATGAACTTTCTGCTG-3’; reverse, 5’-ATAAGCTTTCACCGCCTT-3’) and human glyceraldehyde-3-phosphate dehydrogenase (GAPDH, forward, 5’-GGGCTGCTTTTAACTCTGGT-3’; reverse, 5’-GCAGGTTTTTCTAGACGG-3’). Amplification and detection were performed under the following conditions: Incubation at 95°C for 30 sec, followed by 40 cycles of denaturation at 95°C for 5 sec and subsequent annealing and extension at 60°C for 34 sec. For each sample, GAPDH expression was analyzed to normalize target gene expression. Relative gene expression was calculated using the 2^-ΔΔCq^ method[27]. Each sample was analyzed in triplicate.

### Western blotting

After three washes with cold PBS, total protein was extracted from cells using lysis buffer. The proteins (10 μg) were resolved using 10% SDS-polyacrylamide gel electrophoresis and transferred to polyvinylidene fluoride (PVDF) membranes, which were blocked with 5% nonfat milk in PBS containing Tween-20 (PBS-T) for 2 h at room temperature. The membranes were incubated at 4°C overnight with primary antibodies specific for RUNX2 (1:1000), OCN (1:1000), COL1 (1:500), VEGFR1 (1:1000), Angiogenin (1:500), ERK1/2(1:500), p-ERK1/2(1:500), p38 (1:1,000), p-p38 (1:1,000), JNK (1:500), and p-JNK (1:500). After three washes with PBST (0.5% Tween 20 in PBS), the membranes were incubated with the relevant secondary antibodies (1:1000) for 1 h at 37°C, washed and visualized by Immobilon Western Chemiluminescent HRP Substrate (Millipore) and visualized using the ImageQuantLAS 4000 mini imaging system (General Electrics, USA). Three independent trials of each experiment were carried out. β-actin (1:500) served as an internal control.

### VEGF quantification and HUVECs tube formation assay

The culture supernatant of control and treatment groups was collected from the *in vitro* coculture system after 14 days and assayed to measure the level of VEGF. A human VEGF ELISA kit was used to quantify VEGF in medium from HASCs and HASCs/HAMSCs groups, according to the manufacturer’s instructions. The measured values were expressed as fold changes over that of the control: HASCs treated without HAMSCs.

HUVECs were subjected to the culture supernatant from control and treatment groups to assay the formation of tube-like structures. Six-well culture plates were coated with Matrigel according to the manufacturer’s instructions. After HUVECs were incubated in EBM containing 1% FBS and EGM-2 BulletKit for 6 h and plated onto the layer of Matrigel at a density of 5×10^4^ cells/well, medium was replaced by the culture supernatant from HASCs and HASCs/HAMSCs groups. Matrigel cultures were incubated at 37 C for 24 h. Following rinsing with PBS, tube formation was visualized using an inverted microscope (Carl Zeiss AG, Oberkochen, Germany) and representative network of formed tube structures was randomly photographed five shots per each group.

### Statistical analysis

All the quantitative results were performed using SPSS 13.0 (SPSS Inc., Chicago, IL, USA). Data are presented as the mean ± standard deviation from at least three separate experiments. The Student’s t-test was used to compare data between the two groups. A value of *p* < 0.05 was considered to be statistically significant.

## Results

### HAMSCs promoted HASCs proliferation

**Fig 1:**
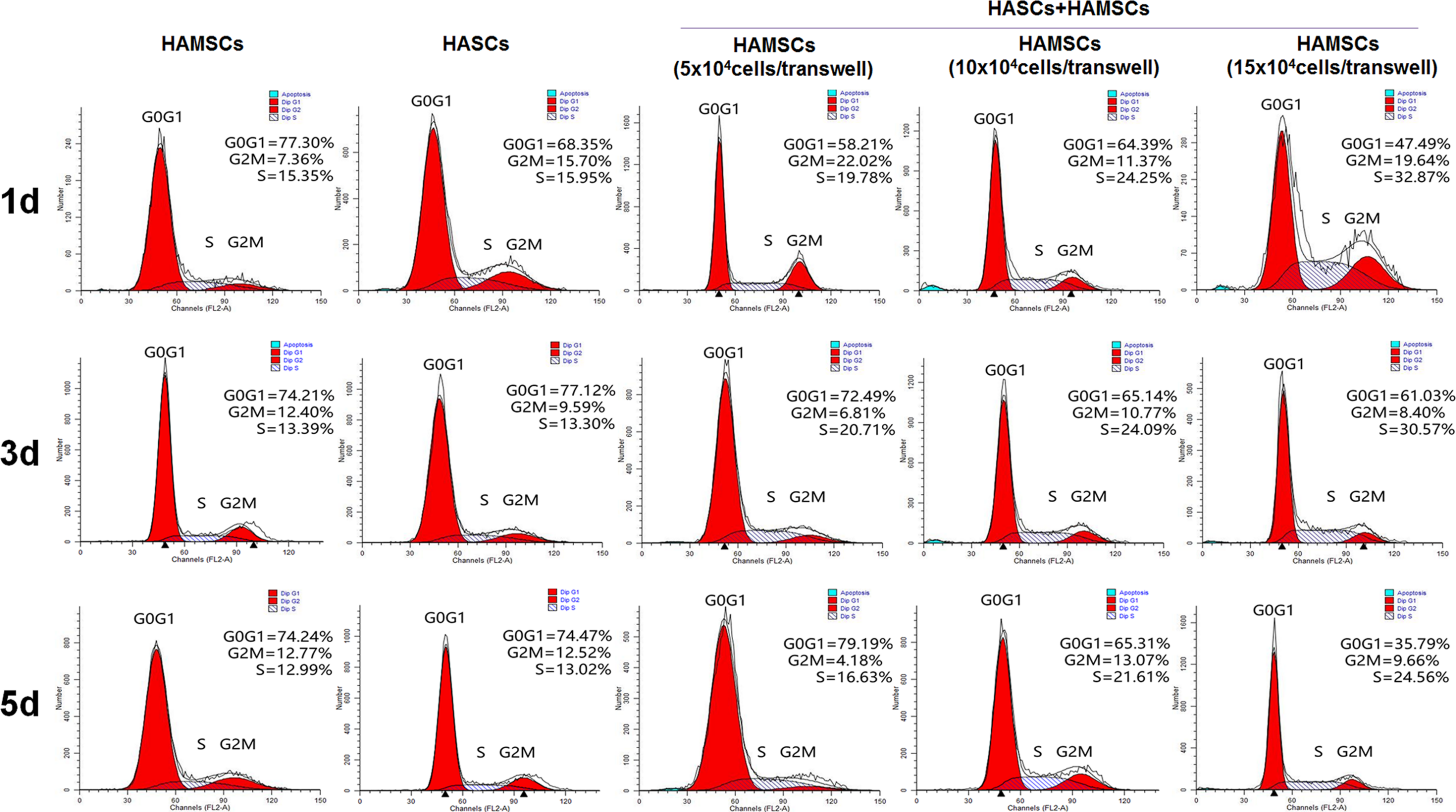
HAMSCs promoted HASCs proliferation. The cell cycle fractions (G0, G1, S, and G2 M phases) of HASCs cultured with or without HAMSCs were determined by flow cytometry at 1, 3 and 5 d.

Cell cycle fractions (G0, G1, S, and G2M phases) were determined by flow cytometry at 1, 3, and 5 days to measure the proliferation of HASCs seeded in the transwell coculture system. There was no significant difference between the proliferation level of HAMSCs and HASCs. However, a statistically increase of S-phase checkpoints with HASCs: HAMSCs ratios in coculture groups compared with the single-culture groups was detected (Fig 1). Our previous studies have confirmed that HAMSCs were capable of enhancing HBMSCs proliferation[20]. The present results further showed that HAMSCs could also accelerate HASCs proliferation in the transwell coculture system.

### HAMSCs promoted ALP activity and extracellular matrix mineralization in HASCs

**Fig 2:**
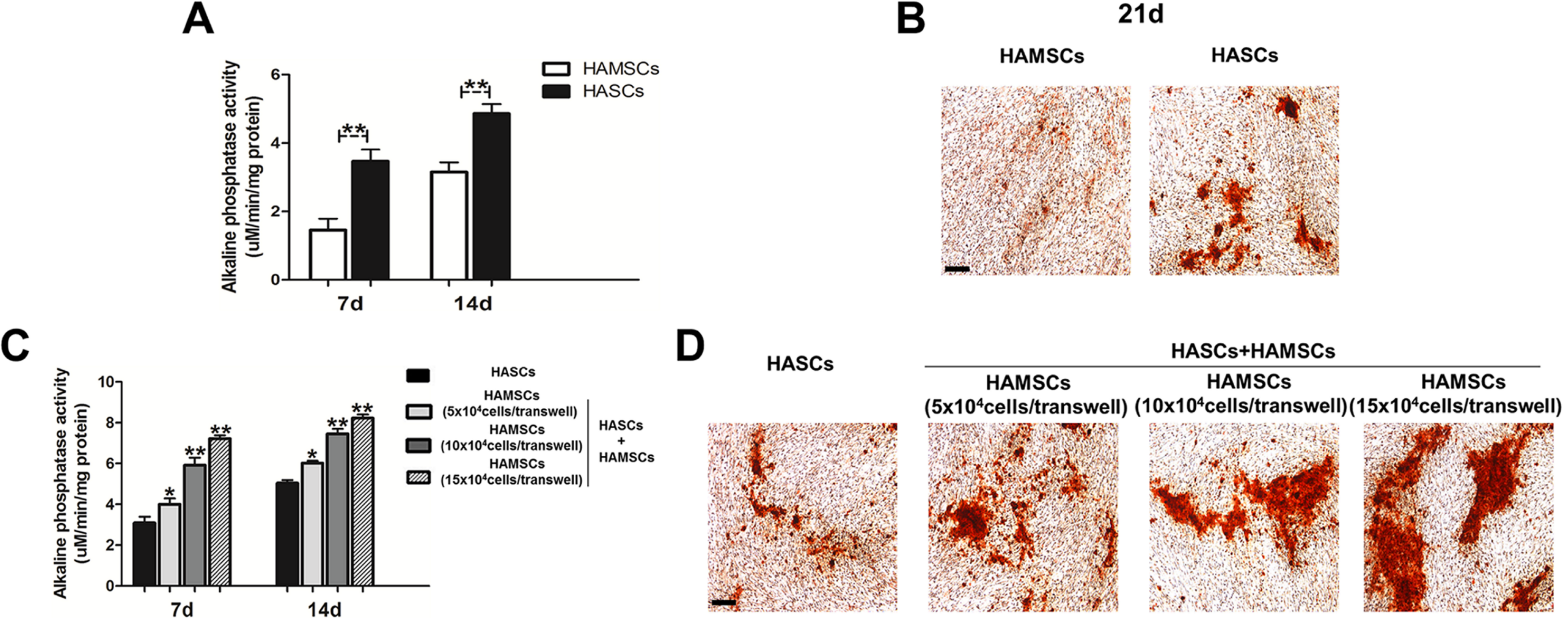
ALP activity and mineralized matrix deposition in HAMSCs, HASCs, and HASCs/HAMSCs groups. **(**A): ALP activity in HAMSCs and HASCs groups was measured at 7 and 14 d using ALP assay kit. (B): Mineralized matrix deposition in HAMSCs and HASCs groups was measured at 21 d by Alizarin red S. (C): ALP activity in HASCs and HASCs/HAMSCs groups was measured at 7 and 14 d using ALP assay kit. (D): Mineralized matrix deposition in HASCs and HASCs/HAMSCs groups was measured at 21 d by Alizarin red S. Scale bar: 300 μm.*P < 0.05 and **P < 0.01 in contrast to the HAMSCs or HASCs groups.

To measure the positive effects of HAMSCs on osteogenic differentiation of HASCs, we investigated ALP activity and extracellular matrix mineralization in HAMSCs, HASCs, and HASCs/HAMSCs groups at different time points. We found that although HAMSCs osteogenesis was much lower than HASCs’, the ALP activity gradually increased in the treatment groups with HASCs: HAMSCs ratio at day 7 and 14, indicating HAMSCs up-regulated the osteoblastic differentiation of HASCs (Fig 2A and 2C).

The HAMSCs, HASCs, and HASCs/HAMSCs culture surfaces stained positively for extracellular matrix was measured after 21 days. HASCs formed more mineralized matrix compared with HAMSCs. Moreover, increased level of mineralization in HASCs/HAMSCs groups was observed after 21 days in comparison with the HASCs single-culture groups. These observations demonstrated that HAMSCs positively promoted the mineralization in HASCs (Fig 2B and 2D).

### HAMSCs promoted osteogenic and angiogenic markers expression in HASCs

**Fig 3:**
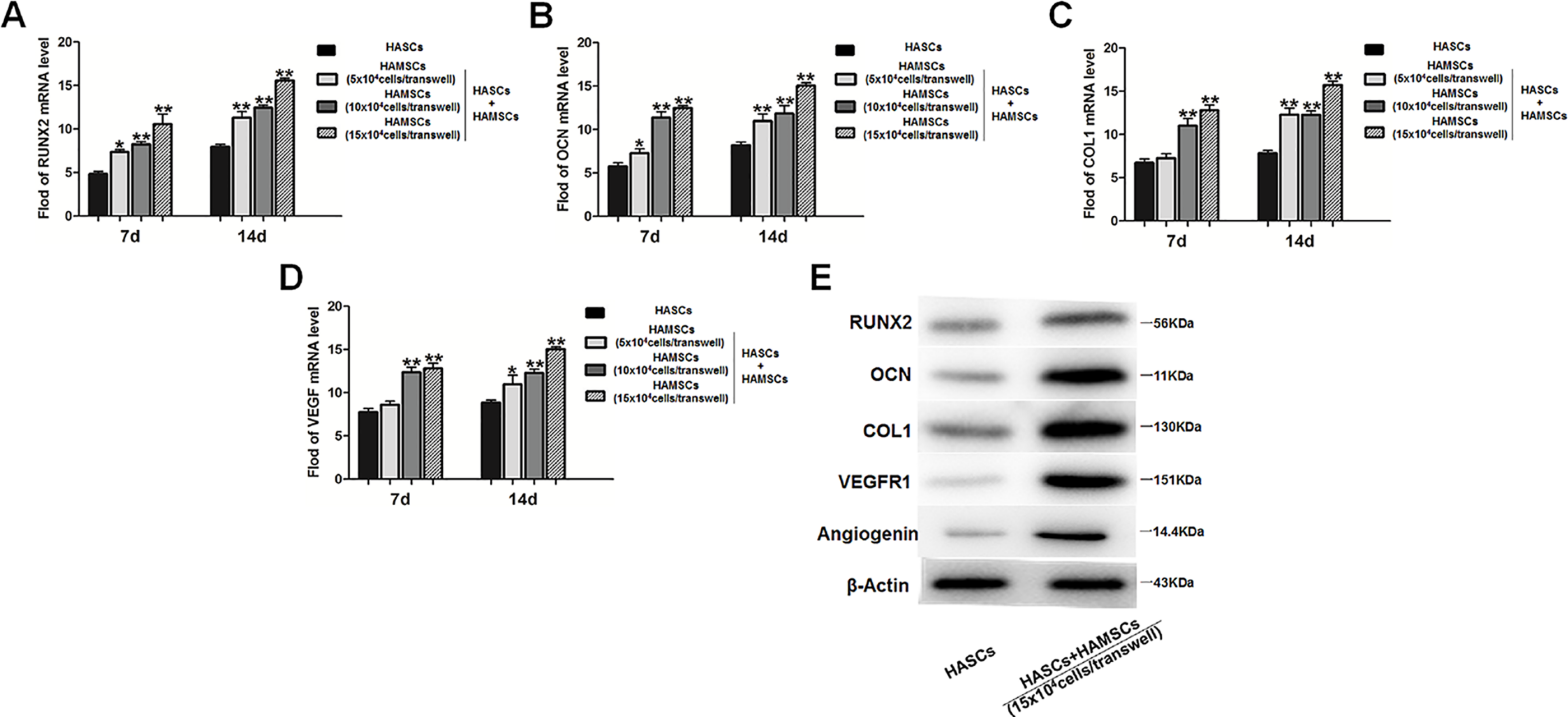
Expression of relative genes and proteins in HASCs cultured with or without HAMSCs. (A, B, C and D): The mRNA expression of RUNX2, OCN, COL1, and VEGF were analyzed by real-time PCR at 14 d. GAPDH were used as the internal control. (E): Protein expression of RUNX2, OCN, COL1, VEGFR1, and Angiogenin were determined by Western blotting at 14 d, β-actin served as an internal control. *P < 0.05 and **P < 0.01 in contrast to the HASCs groups.

Human RUNX2, OCN, and COL1 gene expression were analyzed by real-time PCR in HASCs after 14 days, with and without HAMSCs (Fig 3A, 3B and 3C). Significant higher levels of these osteogenic markers expression were observed in HASCs/HAMSCs groups with increased HASCs: HAMSCs ratio. VEGF, a valuable cytokine engaged in blood vessel formation and angiogenesis, was also up-regulated in HASCs cocultured with HAMSCs (Fig 3D). Western blotting showed the protein expression of RUNX2, OCN, COL1, VEGFR1, and Angiogenin were increased in HASCs/HAMSCs groups (Fig 3E). These results suggested HAMSCs enhanced osteogenic and angiogenic differentiation of HASCs.

### HAMSCs promoted angiogenesis and ERK1/2 phosphorylation in HASCs

**Fig 4:**
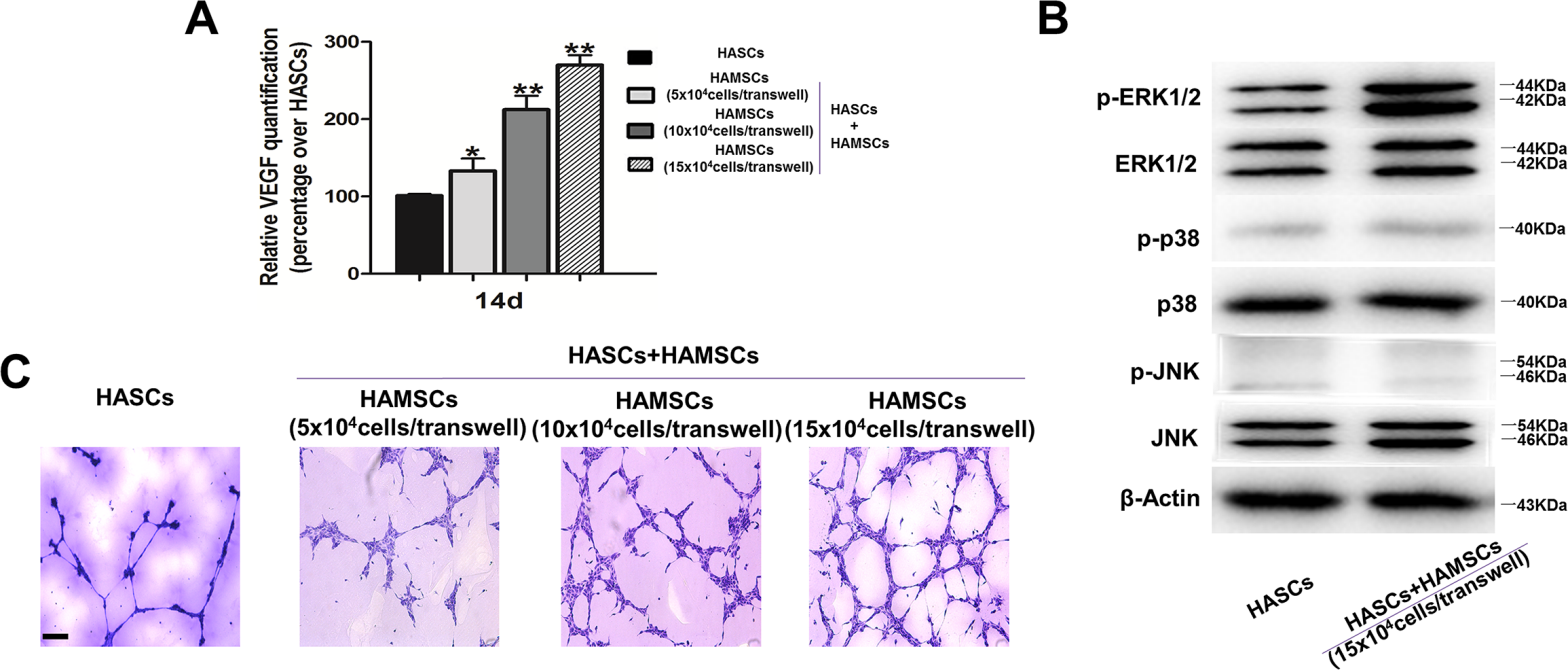
HAMSCs promoted angiogenesis and ERK1/2 phosphorylation in HASCs. (A): The VEGF level in culture supernatant from HASCs and HASCs/HAMSCs groups was measured by VEGF ELISA assay on day 14. (B): Tube formation from HUVECs was detected at 24 h after culture. (C): Protein expression of p-ERK, ERK, p-p38, p38, p-JNK, and JNK were determined by Western blotting at 14 d, β-actin served as an internal control. Scale bar: 300 μm.*P < 0.05 and **P < 0.01 in contrast to the HASCs groups.

The effects of HAMSCs on angiogenesis in HASCs were further measured by VEGF ELISA assay of the culture medium on day 14 and tube formation assay of HUVECs at 24 h after culture. The HASCs/HAMSCs groups secreted significantly higher level of VEGF than HASCs groups, and the VEGF level gradually increased with HASCs: HAMSCs ratio (Fig 4A). The HUVECs tube formation assay demonstrated that the addition of culture supernatant from HASCs/HAMSCs groups significantly increased tube structures formed by HUVECs. Extensive vascular networks were observed and increased with HASCs: HAMSCs ratio in HUVECs subjected to culture medium from HASCs/HAMSCs system (Fig 4C). However, whether the increased VEGF level is related to the interaction between HASCs and HAMSCs should be studied further.

Our previous studies have indicated that MAPK signal pathway is involved in the HAMSCs–droved osteogenic differentiation of HBMSCs[26]. In addition, it was also reported that MAPK signal pathway played an important role in regulating HASCs osteogenic differentiation [28]. Here, to explore the underlying mechanism, we investigated the effects of HAMSCs on MAPK activation in HASCs. In contrast to HASCs single culture groups, HAMSCs activated the ERK1/2 phosphorylation level in HASCs/HAMSCs groups, while neither the p38 nor JNK phosphorylation appeared to be involved (Fig 4B). These findings demonstrated that HAMSCs enhanced ERK1/2 phosphorylation, which might play a role in modulating HASCs osteogenesis and angiogenesis.

## Discussion

The present study identified the valuable effects of HAMSCs on promoting osteogenic and angiogenic potential of HASCs *in vitro*. We found that HAMSCs promoted proliferation, drove osteogenic differentiation, and enhanced angiogenic via secretion increased VEGF in HASCs. Mechanismly, we demonstrated that ERK1/2-MAPK phosphorylation is associated with the osteogenesis and angiogenesis modulated by HAMSCs, while neither the p38 nor JNK phosphorylation appeared to be involved.

The application of MSCs has been widely designed to promote osteogenesis and angiogenesis for regenerating new bone formation in tissue engineering approaches. HBMSCs, osteoblasts (OB), and dental pulp stem cells (DPSCs) have been used [29–31], but most have disadvantages, such as limited availability, high immunogenicity and ethics problems. As an abundant source of stem/progenitor cells, adipose tissue became the focus of considerable interest in regenerative tissue engineering[32]. Among these cells, HASCs gained more attention in recent decades because they can be easily expanded and differentiated into a variety of cells along multiple lineages [33]. Since adipose tissue is abundant and easily access, HASCs exhibit enormous potential for clinical translation into regeneration therapies[34]. Therefore, how to effectively modulate the osteogenic and angiogenic differentiation of HASCs has become an emerging medical problem in bone tissue engineering [35–37].

HAMSCs, isolated from discarded human term placenta, exhibit a potential advantage over other types of MSCs [19]. Our previous findings have showed that HAMSCs were capable of promoting proliferation and osteogenic differentiation of HBMSCs[26], which led us to hypothesize the role of HAMSCs in HASCs-involved new bone formation. The S-phase checkpoints evaluated in HAMSCs, HASCs, and HASCs/HAMSCs groups at 1, 3, and 5 days suggested that HAMSCs promoted HASCs proliferation at early-stage of differentiation. ALP is an early marker of the osteogenic differentiation. Although HAMSCs osteogenesis was much lower than HASCs’, treatment of HAMSCs remarkably up-regulated the ALP activity in HASCs. Increased mineralized nodule formation and calcium deposition activity in HASCs cocultured with HAMSCs indicated the positive effects of HAMSCs on late-stage of osteogenic differentiation [38–40]. RUNX2 is a key transcriptional factor associates with several transcription factors and binds to specific nuclear matrix[41]. It is capable of integrating a variety of organize crucial events during the early-stage osteoblastic differentiation of MSCs [42]. OCN is a marker of mature osteogenesis phenotype reflecting calcium deposition during the late-stage osteoblastic differentiation [43]. COL1 has been extensively investigated as the most important structural protein of natural bone[44], and its main organic component has been shown to display excellent osteoconductive properties *in vitro* and *in vivo*[45, 46]. Collectively, the real-time PCR analysis of RUNX2, OCN, and COL1 gene expression suggested HAMSCs motivated HASCs osteogenic differentiation in both early- and late-stage. Additionally, protein expression showed the same variation, which helped us better comprehend HAMSCs’ mechanism for action in HASCs-involved bone regeneration.

Bone is a vital organism that needs intraosseous vasculature to maintain normal metabolism[47]. A number of studies have demonstrated that rapid revascularization secrete osteogenic factors is necessary for initiating reliable bone formation in bone regeneration [48, 49]. Thus, developing proangiogenic molecules is becoming a key factor in tissue repair.HASCs can secrete a broad range of angiogenic factors, and share equal angiogenic capacity compared with HBMSCs[50]. HAMSCs are capable of acting as stromal cells in the formation, stabilization, and maturation of newly formed vessel [51, 52]. Therefore, we hypothesize the effects of HAMSCs in HASCs-involved neovascularization. VEGF, a cytokine engaged in angiogenesis and osteogenesis, evidently enhances new bone formation in animal fracture models [53, 54]. In this study, higher level of VEGF secretion was found in HASCs/HAMSCs groups than HASCs groups. HUVECs, the most commonly used type of cells for vessel regeneration studies [55], formed abundant vascular networks by treatment with culture supernatant from HASCs/HAMSCs groups. In addition, angiogenesis related gene and protein expression also indicated the positive effects of HAMSCs. Thus, these results support HAMSC as activator in HASCs-involved angiogenesis.

To unveil the underlying molecular mechanisms by which HAMSCs promote HASCs osteogenic and angiogenic differentiation, MAPK signaling, the activation of which is a crucial trigger of MSCs differentiation [56–58], was investigated. Phosphorylation is a remarkable mechanism that modulates the initiating of various factors during osteogenesis and angiogenesis [59–61]. Our previous study determined that ERK1/2 phosphorylation was important for the HAMSCs–droved osteogenic differentiation of HBMSCs [62]. Interestingly, the present study also suggested the p-ERK1/2 level was up-regulated by HAMSCs in HASCs. Previous study reported the ERK1/2-dependent RUNX2 activation was an important regulatory mechanism of osteoblast differentiation [63]. In view of the expression of p-ERK1/2 and RUNX2 were both increased by HAMSCs, the ERK1/2-RUNX2 signaling may represent a suitable therapeutic target for promoting HASCs-involved bone regeneration.

The present study first demonstrates the influence of cocultured HAMSCs in enhancing the osteogenic and angiogenic differentiation of HASCs as well as the potential signal pathway. HAMSCs added to HASCs promoted proliferation, osteogenesis and angiogenesis *in vitro* at the increased HASCs: HAMSCs ratios. The ERK1/2 phosphorylation level, RUNX2 expression, and VEGF secretion are involved in the underlying mechanism. These findings shed light on the key characteristics of HAMSCs and expand the application of HASCs in bone defects reconstruction.

## Supporting information

S1 File Western blotting in Figs 3 and 4. (ZIP)

## References

[1] Llambes F, Silvestre FJ, Caffesse R. Vertical guided bone regeneration with bioabsorbable barriers. Journal of periodontology. 2007;78(10):2036–42. . 18062126 10.1902/jop.2007.070017

[2] Maeda H, Tomokiyo A, Fujii S, Wada N, Akamine A. Promise of periodontal ligament stem cells in regeneration of periodontium. Stem cell research & therapy. 2011;2(4):33 doi: 10.1186/scrt74 ; PubMed Central PMCID: PMC3219064. 21861868 10.1186/scrt74 PMC3219064

[3] Park SH, Wang HL. Clinical significance of incision location on guided bone regeneration: human study. Journal of periodontology. 2007;78(1):47–51. doi: 10.1902/jop.2007.060125 . 17199538 10.1902/jop.2007.060125

[4] Petite H, Viateau V, Bensaid W, Meunier A, de Pollak C, Bourguignon M, et al Tissue-engineered bone regeneration. Nature biotechnology. 2000;18(9):959–63. doi: 10.1038/79449 . 10973216 10.1038/79449

[5] Boccaccini AR, Blaker JJ. Bioactive composite materials for tissue engineering scaffolds. Expert review of medical devices. 2005;2(3):303–17. doi: 10.1586/17434440.2.3.303 . 16288594 10.1586/17434440.2.3.303

[6] Zuk PA, Zhu M, Ashjian P, De Ugarte DA, Huang JI, Mizuno H, et al Human adipose tissue is a source of multipotent stem cells. Molecular biology of the cell. 2002;13(12):4279–95. doi: 10.1091/mbc.E02-02-0105 ; PubMed Central PMCID: PMC138633. 12475952 10.1091/mbc.E02-02-0105 PMC138633

[7] Zuk PA, Zhu M, Mizuno H, Huang J, Futrell JW, Katz AJ, et al Multilineage cells from human adipose tissue: implications for cell-based therapies. Tissue engineering. 2001;7(2):211–28. doi: 10.1089/107632701300062859 . 11304456 10.1089/107632701300062859

[8] Nathan S, Das De S, Thambyah A, Fen C, Goh J, Lee EH. Cell-based therapy in the repair of osteochondral defects: a novel use for adipose tissue. Tissue engineering. 2003;9(4):733–44. doi: 10.1089/107632703768247412 . 13678450 10.1089/107632703768247412

[9] Mizuno H. Adipose-derived stem cells for tissue repair and regeneration: ten years of research and a literature review. Journal of Nippon Medical School = Nippon Ika Daigaku zasshi. 2009;76(2):56–66. . 19443990 10.1272/jnms.76.56

[10] Jin Y, Zhang W, Liu Y, Zhang M, Xu L, Wu Q, et al rhPDGF-BB via ERK pathway osteogenesis and adipogenesis balancing in ADSCs for critical-sized calvarial defect repair. Tissue engineering Part A. 2014;20(23–24):3303–13. doi: 10.1089/ten.TEA.2013.0556 . 24568547 10.1089/ten.TEA.2013.0556

[11] Merfeld-Clauss S, Gollahalli N, March KL, Traktuev DO. Adipose tissue progenitor cells directly interact with endothelial cells to induce vascular network formation. Tissue engineering Part A. 2010;16(9):2953–66. doi: 10.1089/ten.TEA.2009.0635 ; PubMed Central PMCID: PMC2928048. 20486792 10.1089/ten.tea.2009.0635 PMC2928048

[12] Rohringer S, Hofbauer P, Schneider KH, Husa AM, Feichtinger G, Peterbauer-Scherb A, et al Mechanisms of vasculogenesis in 3D fibrin matrices mediated by the interaction of adipose-derived stem cells and endothelial cells. Angiogenesis. 2014;17(4):921–33. doi: 10.1007/s10456-014-9439-0 . 25086616 10.1007/s10456-014-9439-0

[13] Murohara T, Shintani S, Kondo K. Autologous adipose-derived regenerative cells for therapeutic angiogenesis. Current pharmaceutical design. 2009;15(24):2784–90. . 19689349 10.2174/138161209788923796

[14] Mollazadeh S, Neshati V, Fazly Bazzaz BS, Iranshahi M, Mojarrad M, Naderi-Meshkin H, et al Standardized Sophora pachycarpa Root Extract Enhances Osteogenic Differentiation in Adipose-derived Human Mesenchymal Stem Cells. Phytotherapy research: PTR. 2017;31(5):792–800. doi: 10.1002/ptr.5803 . 28337797 10.1002/ptr.5803

[15] Lee JS, Lee JM, Im GI. Electroporation-mediated transfer of Runx2 and Osterix genes to enhance osteogenesis of adipose stem cells. Biomaterials. 2011;32(3):760–8. doi: 10.1016/j.biomaterials.2010.09.042 . 20947160 10.1016/j.biomaterials.2010.09.042

[16] Park KH, Kim H, Moon S, Na K. Bone morphogenic protein-2 (BMP-2) loaded nanoparticles mixed with human mesenchymal stem cell in fibrin hydrogel for bone tissue engineering. Journal of bioscience and bioengineering. 2009;108(6):530–7. doi: 10.1016/j.jbiosc.2009.05.021 . 19914589 10.1016/j.jbiosc.2009.05.021

[17] de Girolamo L, Lucarelli E, Alessandri G, Avanzini MA, Bernardo ME, Biagi E, et al Mesenchymal stem/stromal cells: a new ''cells as drugs'' paradigm. Efficacy and critical aspects in cell therapy. Current pharmaceutical design. 2013;19(13):2459–73. doi: 10.2174/1381612811319130015 ; PubMed Central PMCID: PMC3788322. 23278600 10.2174/1381612811319130015 PMC3788322

[18] Alviano F, Fossati V, Marchionni C, Arpinati M, Bonsi L, Franchina M, et al Term Amniotic membrane is a high throughput source for multipotent Mesenchymal Stem Cells with the ability to differentiate into endothelial cells in vitro. BMC developmental biology. 2007;7:11 doi: 10.1186/1471-213X-7-11 ; PubMed Central PMCID: PMC1810523. 17313666 10.1186/1471-213X-7-11 PMC1810523

[19] Leyva-Leyva M, Barrera L, Lopez-Camarillo C, Arriaga-Pizano L, Orozco-Hoyuela G, Carrillo-Casas EM, et al Characterization of mesenchymal stem cell subpopulations from human amniotic membrane with dissimilar osteoblastic potential. Stem cells and development. 2013;22(8):1275–87. doi: 10.1089/scd.2012.0359 . 23211052 10.1089/scd.2012.0359

[20] Wang Y, Yin Y, Jiang F, Chen N. Human amnion mesenchymal stem cells promote proliferation and osteogenic differentiation in human bone marrow mesenchymal stem cells. Journal of molecular histology. 2015;46(1):13–20. doi: 10.1007/s10735-014-9600-5 . 25432786 10.1007/s10735-014-9600-5

[21] Li Y, Guo L, Ahn HS, Kim MH, Kim SW. Amniotic mesenchymal stem cells display neurovascular tropism and aid in the recovery of injured peripheral nerves. Journal of cellular and molecular medicine. 2014;18(6):1028–34. doi: 10.1111/jcmm.12249 ; PubMed Central PMCID: PMC4508143. 24708439 10.1111/jcmm.12249 PMC4508143

[22] Kim SW, Zhang HZ, Kim CE, An HS, Kim JM, Kim MH. Amniotic mesenchymal stem cells have robust angiogenic properties and are effective in treating hindlimb ischaemia. Cardiovascular research. 2012;93(3):525–34. doi: 10.1093/cvr/cvr328 . 22155484 10.1093/cvr/cvr328

[23] Zhang D, Jiang M, Miao D. Transplanted human amniotic membrane-derived mesenchymal stem cells ameliorate carbon tetrachloride-induced liver cirrhosis in mouse. PloS one. 2011;6(2):e16789 doi: 10.1371/journal.pone.0016789 ; PubMed Central PMCID: PMC3033905. 21326862 10.1371/journal.pone.0016789 PMC3033905

[24] Soncini M, Vertua E, Gibelli L, Zorzi F, Denegri M, Albertini A, et al Isolation and characterization of mesenchymal cells from human fetal membranes. Journal of tissue engineering and regenerative medicine. 2007;1(4):296–305. doi: 10.1002/term.40 . 18038420 10.1002/term.40

[25] Jiang F, Ma J, Liang Y, Niu Y, Chen N, Shen M. Amniotic Mesenchymal Stem Cells Can Enhance Angiogenic Capacity via MMPs In Vitro and In Vivo. BioMed research international. 2015;2015:324014 doi: 10.1155/2015/324014 ; PubMed Central PMCID: PMC4600487. 26491665 10.1155/2015/324014 PMC4600487

[26] Wang Y, Jiang F, Liang Y, Shen M, Chen N. Human Amnion-Derived Mesenchymal Stem Cells Promote Osteogenic Differentiation in Human Bone Marrow Mesenchymal Stem Cells by Influencing the ERK1/2 Signaling Pathway. Stem cells international. 2016;2016:4851081 doi: 10.1155/2016/4851081 ; PubMed Central PMCID: PMC4677248. 26697075 10.1155/2016/4851081 PMC4677248

[27] Livak KJ, Schmittgen TD. Analysis of relative gene expression data using real-time quantitative PCR and the 2(-Delta Delta C(T)) Method. Methods. 2001;25(4):402–8. doi: 10.1006/meth.2001.1262 . 11846609 10.1006/meth.2001.1262

[28] Liu Q, Cen L, Zhou H, Yin S, Liu G, Liu W, et al The role of the extracellular signal-related kinase signaling pathway in osteogenic differentiation of human adipose-derived stem cells and in adipogenic transition initiated by dexamethasone. Tissue engineering Part A. 2009;15(11):3487–97. doi: 10.1089/ten.TEA.2009.0175 . 19438323 10.1089/ten.TEA.2009.0175

[29] Kim SH, Kim KH, Seo BM, Koo KT, Kim TI, Seol YJ, et al Alveolar bone regeneration by transplantation of periodontal ligament stem cells and bone marrow stem cells in a canine peri-implant defect model: a pilot study. Journal of periodontology. 2009;80(11):1815–23. doi: 10.1902/jop.2009.090249 . 19905951 10.1902/jop.2009.090249

[30] Wang S, Zhang Z, Zhao J, Zhang X, Sun X, Xia L, et al Vertical alveolar ridge augmentation with beta-tricalcium phosphate and autologous osteoblasts in canine mandible. Biomaterials. 2009;30(13):2489–98. doi: 10.1016/j.biomaterials.2008.12.067 . 19147220 10.1016/j.biomaterials.2008.12.067

[31] Zhao J, Zhang Z, Wang S, Sun X, Zhang X, Chen J, et al Apatite-coated silk fibroin scaffolds to healing mandibular border defects in canines. Bone. 2009;45(3):517–27. doi: 10.1016/j.bone.2009.05.026 ; PubMed Central PMCID: PMC2828815. 19505603 10.1016/j.bone.2009.05.026 PMC2828815

[32] Ayatollahi M, Talaei-Khozani T, Razmkhah M. Growth suppression effect of human mesenchymal stem cells from bone marrow, adipose tissue, and Wharton's jelly of umbilical cord on PBMCs. Iranian journal of basic medical sciences. 2016;19(2):145–53. ; PubMed Central PMCID: PMC4818361. 27081458 PMC4818361

[33] Bosnakovski D, Mizuno M, Kim G, Takagi S, Okumura M, Fujinaga T. Isolation and multilineage differentiation of bovine bone marrow mesenchymal stem cells. Cell and tissue research. 2005;319(2):243–53. doi: 10.1007/s00441-004-1012-5 . 15654654 10.1007/s00441-004-1012-5

[34] Gimble JM, Katz AJ, Bunnell BA. Adipose-derived stem cells for regenerative medicine. Circulation research. 2007;100(9):1249–60. doi: 10.1161/01.RES.0000265074.83288.09 . 17495232 10.1161/01.RES.0000265074.83288.09 PMC5679280

[35] Bodle JC, Hanson AD, Loboa EG. Adipose-derived stem cells in functional bone tissue engineering: lessons from bone mechanobiology. Tissue engineering Part B, Reviews. 2011;17(3):195–211. doi: 10.1089/ten.TEB.2010.0738 ; PubMed Central PMCID: PMC3098956. 21338267 10.1089/ten.teb.2010.0738 PMC3098956

[36] Tsuji W, Rubin JP, Marra KG. Adipose-derived stem cells: Implications in tissue regeneration. World journal of stem cells. 2014;6(3):312–21. doi: 10.4252/wjsc.v6.i3.312 ; PubMed Central PMCID: PMC4131273. 25126381 10.4252/wjsc.v6.i3.312 PMC4131273

[37] Ruetze M, Richter W. Adipose-derived stromal cells for osteoarticular repair: trophic function versus stem cell activity. Expert reviews in molecular medicine. 2014;16:e9 doi: 10.1017/erm.2014.9 ; PubMed Central PMCID: PMC4017835. 24810570 10.1017/erm.2014.9 PMC4017835

[38] Bahlous A, Kalai E, Hadj Salah M, Bouzid K, Zerelli L. [Biochemical markers of bone remodeling: recent data of their applications in managing postmenopausal osteoporosis]. La Tunisie medicale. 2006;84(11):751–7. . 17294906

[39] Balcerzak M, Hamade E, Zhang L, Pikula S, Azzar G, Radisson J, et al The roles of annexins and alkaline phosphatase in mineralization process. Acta biochimica Polonica. 2003;50(4):1019–38. 0350041019. doi: 0350041019 . 14739992

[40] Thangakumaran S, Sudarsan S, Arun KV, Talwar A, James JR. Osteoblast response (initial adhesion and alkaline phosphatase activity) following exposure to a barrier membrane/enamel matrix derivative combination. Indian journal of dental research: official publication of Indian Society for Dental Research. 2009;20(1):7–12. . 19336852 10.4103/0970-9290.49048

[41] Komori T. Regulation of osteoblast differentiation by transcription factors. Journal of cellular biochemistry. 2006;99(5):1233–9. doi: 10.1002/jcb.20958 . 16795049 10.1002/jcb.20958

[42] Phimphilai M, Zhao Z, Boules H, Roca H, Franceschi RT. BMP signaling is required for RUNX2-dependent induction of the osteoblast phenotype. Journal of bone and mineral research: the official journal of the American Society for Bone and Mineral Research. 2006;21(4):637–46. doi: 10.1359/jbmr.060109 ; PubMed Central PMCID: PMC2435171. 16598384 10.1359/JBMR.060109 PMC2435171

[43] Xia L, Yin Z, Mao L, Wang X, Liu J, Jiang X, et al Akermanite bioceramics promote osteogenesis, angiogenesis and suppress osteoclastogenesis for osteoporotic bone regeneration. Scientific reports. 2016;6:22005 doi: 10.1038/srep22005 ; PubMed Central PMCID: PMC4766478. 26911441 10.1038/srep22005 PMC4766478

[44] Yang X, Shah JD, Wang H. Nanofiber enabled layer-by-layer approach toward three-dimensional tissue formation. Tissue engineering Part A. 2009;15(4):945–56. doi: 10.1089/ten.tea.2007.0280 . 18788981 10.1089/ten.tea.2007.0280

[45] Kobayashi K, Anada T, Handa T, Kanda N, Yoshinari M, Takahashi T, et al Osteoconductive property of a mechanical mixture of octacalcium phosphate and amorphous calcium phosphate. ACS applied materials & interfaces. 2014;6(24):22602–11. doi: 10.1021/am5067139 . 25478703 10.1021/am5067139

[46] Mushahary D, Wen C, Kumar JM, Lin J, Harishankar N, Hodgson P, et al Collagen type-I leads to in vivo matrix mineralization and secondary stabilization of Mg-Zr-Ca alloy implants. Colloids and surfaces B, Biointerfaces. 2014;122:719–28. doi: 10.1016/j.colsurfb.2014.08.005 . 25179112 10.1016/j.colsurfb.2014.08.005

[47] Santos MI, Reis RL. Vascularization in bone tissue engineering: physiology, current strategies, major hurdles and future challenges. Macromolecular bioscience. 2010;10(1):12–27. doi: 10.1002/mabi.200900107 . 19688722 10.1002/mabi.200900107

[48] Kanczler JM, Oreffo RO. Osteogenesis and angiogenesis: the potential for engineering bone. European cells & materials. 2008;15:100–14. . 18454418 10.22203/ecm.v015a08

[49] Carano RA, Filvaroff EH. Angiogenesis and bone repair. Drug discovery today. 2003;8(21):980–9. . 14643161 10.1016/s1359-6446(03)02866-6

[50] Hsiao ST, Asgari A, Lokmic Z, Sinclair R, Dusting GJ, Lim SY, et al Comparative analysis of paracrine factor expression in human adult mesenchymal stem cells derived from bone marrow, adipose, and dermal tissue. Stem cells and development. 2012;21(12):2189–203. doi: 10.1089/scd.2011.0674 ; PubMed Central PMCID: PMC3411362. 22188562 10.1089/scd.2011.0674 PMC3411362

[51] Verseijden F, Posthumus-van Sluijs SJ, Pavljasevic P, Hofer SO, van Osch GJ, Farrell E. Adult human bone marrow- and adipose tissue-derived stromal cells support the formation of prevascular-like structures from endothelial cells in vitro. Tissue engineering Part A. 2010;16(1):101–14. doi: 10.1089/ten.TEA.2009.0106 . 19642855 10.1089/ten.TEA.2009.0106

[52] Konig J, Huppertz B, Desoye G, Parolini O, Frohlich JD, Weiss G, et al Amnion-derived mesenchymal stromal cells show angiogenic properties but resist differentiation into mature endothelial cells. Stem cells and development. 2012;21(8):1309–20. doi: 10.1089/scd.2011.0223 . 21762016 10.1089/scd.2011.0223

[53] Kmiecik G, Niklinska W, Kuc P, Pancewicz-Wojtkiewicz J, Fil D, Karwowska A, et al Fetal membranes as a source of stem cells. Advances in medical sciences. 2013;58(2):185–95. doi: 10.2478/ams-2013-0007 . 24327530 10.2478/ams-2013-0007

[54] Cui F, Wang X, Liu X, Dighe AS, Balian G, Cui Q. VEGF and BMP-6 enhance bone formation mediated by cloned mouse osteoprogenitor cells. Growth factors. 2010;28(5):306–17. doi: 10.3109/08977194.2010.484423 . 20497064 10.3109/08977194.2010.484423

[55] Alard JE, Dueymes M, Mageed RA, Saraux A, Youinou P, Jamin C. Mitochondrial heat shock protein (HSP) 70 synergizes with HSP60 in transducing endothelial cell apoptosis induced by anti-HSP60 autoantibody. FASEB journal: official publication of the Federation of American Societies for Experimental Biology. 2009;23(8):2772–9. doi: 10.1096/fj.08-128785 . 19346294 10.1096/fj.08-128785

[56] Hu N, Feng C, Jiang Y, Miao Q, Liu H. Regulative Effect of Mir-205 on Osteogenic Differentiation of Bone Mesenchymal Stem Cells (BMSCs): Possible Role of SATB2/Runx2 and ERK/MAPK Pathway. International journal of molecular sciences. 2015;16(5):10491–506. doi: 10.3390/ijms160510491 ; PubMed Central PMCID: PMC4463658. 25961955 10.3390/ijms160510491 PMC4463658

[57] Herbert BA, Valerio MS, Gaestel M, Kirkwood KL. Sexual Dimorphism in MAPK-Activated Protein Kinase-2 (MK2) Regulation of RANKL-Induced Osteoclastogenesis in Osteoclast Progenitor Subpopulations. PloS one. 2015;10(5):e0125387 doi: 10.1371/journal.pone.0125387 ; PubMed Central PMCID: PMC4422514. 25946081 10.1371/journal.pone.0125387 PMC4422514

[58] Qin L, Tang B, Deng B, Mohan C, Wu T, Peng A. Extracellular regulated protein kinases play a key role via bone morphogenetic protein 4 in high phosphate-induced endothelial cell apoptosis. Life sciences. 2015;131:37–43. doi: 10.1016/j.lfs.2015.03.017 . 25896660 10.1016/j.lfs.2015.03.017

[59] Kim BG, Kim HJ, Park HJ, Kim YJ, Yoon WJ, Lee SJ, et al Runx2 phosphorylation induced by fibroblast growth factor-2/protein kinase C pathways. Proteomics. 2006;6(4):1166–74. doi: 10.1002/pmic.200500289 . 16421932 10.1002/pmic.200500289

[60] Selvamurugan N, Pulumati MR, Tyson DR, Partridge NC. Parathyroid hormone regulation of the rat collagenase-3 promoter by protein kinase A-dependent transactivation of core binding factor alpha1. The Journal of biological chemistry. 2000;275(7):5037–42. . 10671545 10.1074/jbc.275.7.5037

[61] Qiao M, Shapiro P, Kumar R, Passaniti A. Insulin-like growth factor-1 regulates endogenous RUNX2 activity in endothelial cells through a phosphatidylinositol 3-kinase/ERK-dependent and Akt-independent signaling pathway. The Journal of biological chemistry. 2004;279(41):42709–18. doi: 10.1074/jbc.M404480200 . 15304489 10.1074/jbc.M404480200

[62] Wang Y, Ma J, Du Y, Miao J, Chen N. Human Amnion-Derived Mesenchymal Stem Cells Protect Human Bone Marrow Mesenchymal Stem Cells against Oxidative Stress-Mediated Dysfunction via ERK1/2 MAPK Signaling. Molecules and cells. 2016;39(3):186–94. doi: 10.14348/molcells.2016.2159 ; PubMed Central PMCID: PMC4794600. 26743906 10.14348/molcells.2016.2159 PMC4794600

[63] Jeong HM, Han EH, Jin YH, Choi YH, Lee KY, Jeong HG. Xanthohumol from the hop plant stimulates osteoblast differentiation by RUNX2 activation. Biochemical and biophysical research communications. 2011;409(1):82–9. doi: 10.1016/j.bbrc.2011.04.113 . 21565172 10.1016/j.bbrc.2011.04.113

