# Supplementary figures and images for: Human amnion-derived mesenchymal stem cells promote osteogenic and angiogenic differentiation of human adipose-derived stem cells
Source: PLoS One. 2017 Oct 11;12(10):e0186253. doi: 10.1371/journal.pone.0186253 (PMC5636128; doi:10.1371/journal.pone.0186253)

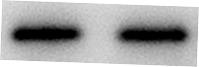

Supplement: S1 File — (ZIP) [file pone.0186253.s001.zip › Supporting Information files/S1 File/Figure.3 WB/ACTIN.tif]

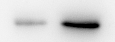

Supplement: S1 File — (ZIP) [file pone.0186253.s001.zip › Supporting Information files/S1 File/Figure.3 WB/Angiogenin.TIF]

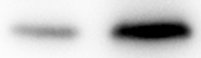

Supplement: S1 File — (ZIP) [file pone.0186253.s001.zip › Supporting Information files/S1 File/Figure.3 WB/OCN.tif]

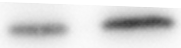

Supplement: S1 File — (ZIP) [file pone.0186253.s001.zip › Supporting Information files/S1 File/Figure.3 WB/RUNX2.tif]

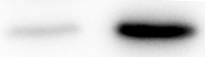

Supplement: S1 File — (ZIP) [file pone.0186253.s001.zip › Supporting Information files/S1 File/Figure.3 WB/VEGFR1.tif]

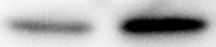

Supplement: S1 File — (ZIP) [file pone.0186253.s001.zip › Supporting Information files/S1 File/Figure.3 WB/col1.tif]

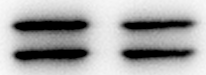

Supplement: S1 File — (ZIP) [file pone.0186253.s001.zip › Supporting Information files/S1 File/Figure.4 WB/ERK.tif]

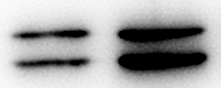

Supplement: S1 File — (ZIP) [file pone.0186253.s001.zip › Supporting Information files/S1 File/Figure.4 WB/P-ERK.tif]

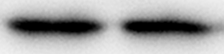

Supplement: S1 File — (ZIP) [file pone.0186253.s001.zip › Supporting Information files/S1 File/Figure.4 WB/actin.tif]

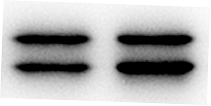

Supplement: S1 File — (ZIP) [file pone.0186253.s001.zip › Supporting Information files/S1 File/Figure.4 WB/jnk.tif]

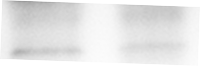

Supplement: S1 File — (ZIP) [file pone.0186253.s001.zip › Supporting Information files/S1 File/Figure.4 WB/p-jnk.tif]

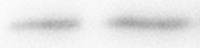

Supplement: S1 File — (ZIP) [file pone.0186253.s001.zip › Supporting Information files/S1 File/Figure.4 WB/p-p38.tif]

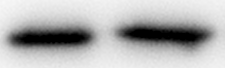

Supplement: S1 File — (ZIP) [file pone.0186253.s001.zip › Supporting Information files/S1 File/Figure.4 WB/p38.tif]
